# The key cyclic electron flow protein PGR5 associates with cytochrome *b*_6_*f*, and its function is partially influenced by the LHCII state transition

**DOI:** 10.1038/s41438-021-00460-y

**Published:** 2021-03-04

**Authors:** Xinyi Wu, Jianqiang Wu, Yu Wang, Meiwen He, Mingming He, Weikang Liu, Sheng Shu, Jin Sun, Shirong Guo

**Affiliations:** 1 College of Horticulture, Nanjing Agricultural University, Nanjing, 210095 China; 2 Nanjing Agricultural University (Suqian) Academy of Protected Horticulture, Jiangsu Suqian, 223800 China

**Keywords:** Photosynthesis, Plant stress responses

## Abstract

In plants and algae, PGR5-dependent cyclic electron flow (CEF) is an important regulator of acclimation to fluctuating environments, but how PGR5 participates in CEF is unclear. In this work, we analyzed two PGR5s in cucumber (*Cucumis sativus* L.) under different conditions and found that CsPGR5a played the dominant role in PGR5-dependent CEF. The results of yeast two-hybrid, biomolecular fluorescence complementation (BiFC), blue native PAGE, and coimmunoprecipitation (CoIP) assays showed that PGR5a interacted with PetC, Lhcb3, and PsaH. Furthermore, the intensity of the interactions was dynamic during state transitions, and the abundance of PGR5 attached to cyt *b*_6_*f* decreased during the transition from state 1 to state 2, which revealed that the function of PGR5a is related to the state transition. We proposed that PGR5 is a small mobile protein that functions when attached to protein complexes.

Two PGR5s are present in some species of algae and higher plants, and CsPGR5a plays the dominant role in PGR5-dependent cyclic electron flow in cucumber. PGR5 is a small and mobile protein that functions when attached to protein complexes. In this study, the function of PGR5 was found to be partially related to the state transition.

## Introduction

Photosynthesis, which is essential to life on Earth, involves the capture and transformation of light energy into chemical energy. During light-dependent photosynthesis, linear electron flow (LEF) driven by PSII and PSI is the dominant electron transport process, which yields ATP and NADPH for CO_2_ assimilation, while cyclic electron flow (CEF) around PSI is an accessory electron transport process that is driven by PSI alone and supplies extra ATP without causing an accumulation of NADPH. CEF was proposed by the Arnon group^1^ and is defined as the rerouting of electrons from the PSI acceptor side back to the donor side. After a long period of debate, a consensus has emerged that CEF plays a general and important role in the regulation of photosynthesis, especially under fluctuating conditions^2–4^. Nonetheless, the exact driving mechanism of CEF is still poorly understood.

Two CEF routes that are capable of reducing plastoquinone (PQ) and shuttling electrons from PSI have been proposed: an NDH-dependent pathway and a PGR5/PGRL1-dependent pathway. The latter is thought to be the primary CEF route in plants. PGR5/PGRL1 was discovered in mutagenized Arabidopsis plants that presented distinctly low ΔpH values across the thylakoid membrane and low steady-state qE values; thus, the name “proton gradient regulation” has been used^5,6^. Moreover, PGRL1 has been proposed to be the elusive ferredoxin (Fd)-PQ reductase in Arabidopsis^7^. By using sucrose density gradient (SDG) centrifugation, Steinbeck et al.^8^ isolated a PSI-LHCI-cyt b_6_f supercomplex comprising several small proteins, such as PGRL1, FNR, ANR1, and CAS, that contributes to CEF in *Chlamydomonas reinhardtii*; however, this kind of supercomplex has not yet been found in angiosperms. Unfortunately, PGR5 has not been found in any purified supercomplex thus far, leading to uncertainty regarding the role of PGR5 in the CEF process, and whether PGR5 directly or indirectly transfers electrons from PSI acceptors to the PQ pool is highly debated^4^.

CEF shares many electron transfer components with LEF, such as cyt *b*_6_*f*, PQ, PC, FNR, and Fd; thus, fine tuning of LEF and CEF is vital^2^. However, the identity of the link between CEF and LEF and how these shared components contribute to the two different electron transport pathways are unclear. In this context, the photosynthetic electron chain may undergo a massive reorganization on behalf of the state transition, which may reflect a simple and intriguing model for the switch between LEF and CEF^9^. Nevertheless, Takahashi et al.^10^ proposed that CEF is independent of the state transition but is redox controlled. Therefore, the relation between CEF and the state transition is still unknown due to the different study objectives and species used; in addition, because evolution occurs in response to a variety of growth environments, the results acquired from one species cannot always be generalized to other species. Thus far, most analyses of CEF have focused on the unicellular green alga *Chlamydomonas reinhardtii* and Arabidopsis. In this work, we analyzed PGR5 in cucumber and found that it closely interacts with PetC (a subunit of the cyt *b*_6_*f* complex). Furthermore, analysis of the interaction between PGR5 and Lhcb3/PsaH revealed that CEF is partially implicated in the state transition.

## Results

### Identification of two PGR5 members in the cucumber genome database

We identified PGR5 genes in the cucumber genome database (http://cucurbitgenomics.org/). Interestingly, two putative *PGR5* sequences are present in cucumber, which are located on chromosome 2 and chromosome 3, while there is just one PGR5 sequence in Arabidopsis. The two *PGR5* sequences were named *PGR5a* (CsaV3_2G012500) and *PGR5b* (CsaV3_3G011780), and we found that the similarity between them was high (Table S1, Fig. S3). Alignments among the sequences from 11 species showed that the mature part of PGR5 (the last 73 amino acid residues)^11,12^ was highly conserved (Fig. S1).

A phylogenetic tree of the sequences from 54 species showed that plant-type PGR5s were widely distributed in organisms ranging from cyanobacteria and algae to plants (Fig. S2). In prokaryotic organisms, there is only one PGR5 protein that regulates CEF, but another PGR5 protein was found to be present in some algae and in a large proportion of higher plants.

The two PGR5s in cucumber are located in chloroplasts (Fig. S4a), and both presented similar gene expression patterns in different organs. High gene expression levels were detected in the leaves and tendrils, while the gene expression levels in the flowers were low. The transcript levels of *CsPGR5a* in young leaves, mature leaves, old leaves, and tendrils were ~6-, 5-, 1-, and 2-fold higher than those of *CsPGR5b*, respectively, implying that *CsPGR5a* plays a more dominant role in cucumber plants (Fig. S4b).

**Fig. 1:**
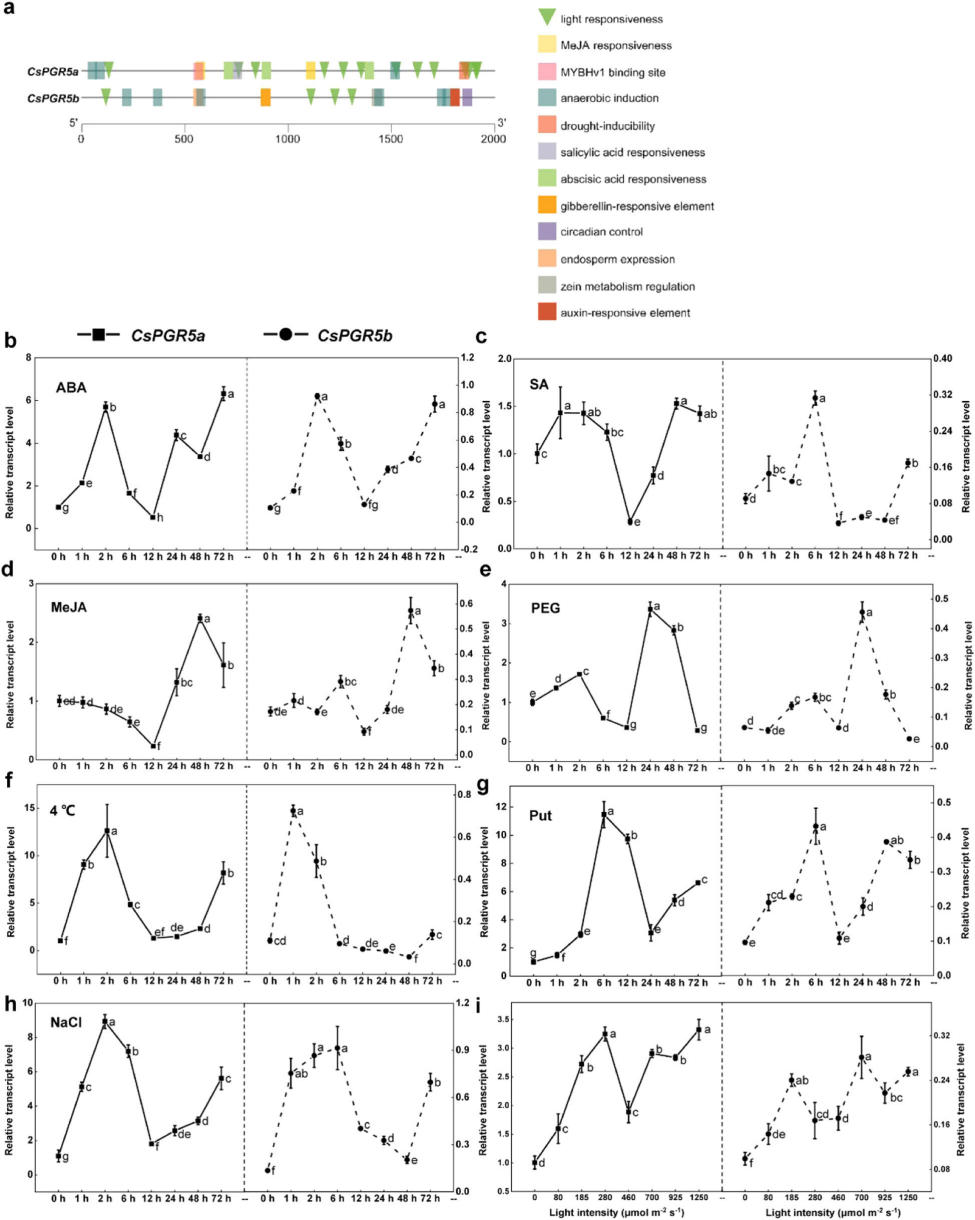
CsPGR5a and CsPGR5b are involved in the responses to multiple hormones and abiotic stress. **a** Main cis-acting elements in the promoters of cucumber *PGR5* genes, as predicted by PlantCARE. **b**–**h** Expression patterns of the *CsPGR5a* and *CsPGR5b* genes in cucumber leaves under different hormone treatments, abiotic stresses and Put treatment. Every morning when the lights were turned on (at 0, 24, 48 h), the leaves were sprayed with 100 μM ABA, 100 μM salicylic acid (SA), 100 μM methyl jasmonate (MeJA) or 8mM Put; treated with 20% polyethylene glycol (PEG) 6000 to mimic drought stress; subjected to 4 °C to induce cold stress; or treated with 100 mM NaCl to induce salt stress. **i** Relative expression levels of the *CsPGR5a* and *CsPGR5b* genes under different light conditions. The label “0” means that the seedlings were fully dark-adapted. The relative gene expression levels were normalized to the level of CsPGR5a at 0h or at 0 light intensity. The different letters indicate significant differences between treatments (*P* < 0.05) according to Tukey’s test

To better understand the functions of the two PGR5s in cucumber, we analyzed the main *cis*-elements in the promoters of cucumber *PGR5* genes via online resources (http://bioinformatics.psb.ugent.be/webtools/plantcare/html/). The results revealed 31 and 15 cis-elements related to hormones and abiotic stress responses in the promoters of *CsPGR5a* and *CsPGR5b*, respectively (Table S2, Fig. 1a). Except for four light-responsive and five anaerobic induction elements, *CsPGR5b* had completely different cis-elements involved in the gibberellin response, circadian control, endosperm expression, zein metabolism regulation, anoxic-specific induction and auxin responses in its promoter, indicating that, compared with CsPGR5a, CsPGR5b responds to different elicitors in some respect. We then subjected cucumber seedlings to exogenous abscisic acid (ABA) treatment, salicylic acid (SA) treatment, methyl jasmonate (MeJA) treatment, putrescine (Put) treatment, 20% polyethylene glycol (PEG) treatment (to mimic drought stress), cold (4 °C), salt stress (100 mM NaCl), and different light intensities. Quantitative real-time PCR (qRT-PCR) results showed that both *CsPGR5a* and *CsPGR5b* positively responded to various hormones, abiotic stress, and Put. The relative transcript levels of both *CsPGR5* genes rapidly increased within 6 h; interestingly, in the following 6 h, the expression levels of the CsPGR5s returned to the baseline levels, which may have been due to the short-term adaptation. After 12 h, the *CsPGR5s* expression levels increased again. Overall, CsPGR5b exhibited a response curve similar to that of *CsPGR5a*, but its induction level was lower than that of *CsPGR5a* (Fig. 1b–i). Given its dominant role in PGR5-dependent CEF, *CsPGR5a* was further analyzed in subsequent experiments.

### PGR5a interacts with PetC, Lhcb3, and PsaH

**Fig. 2:**
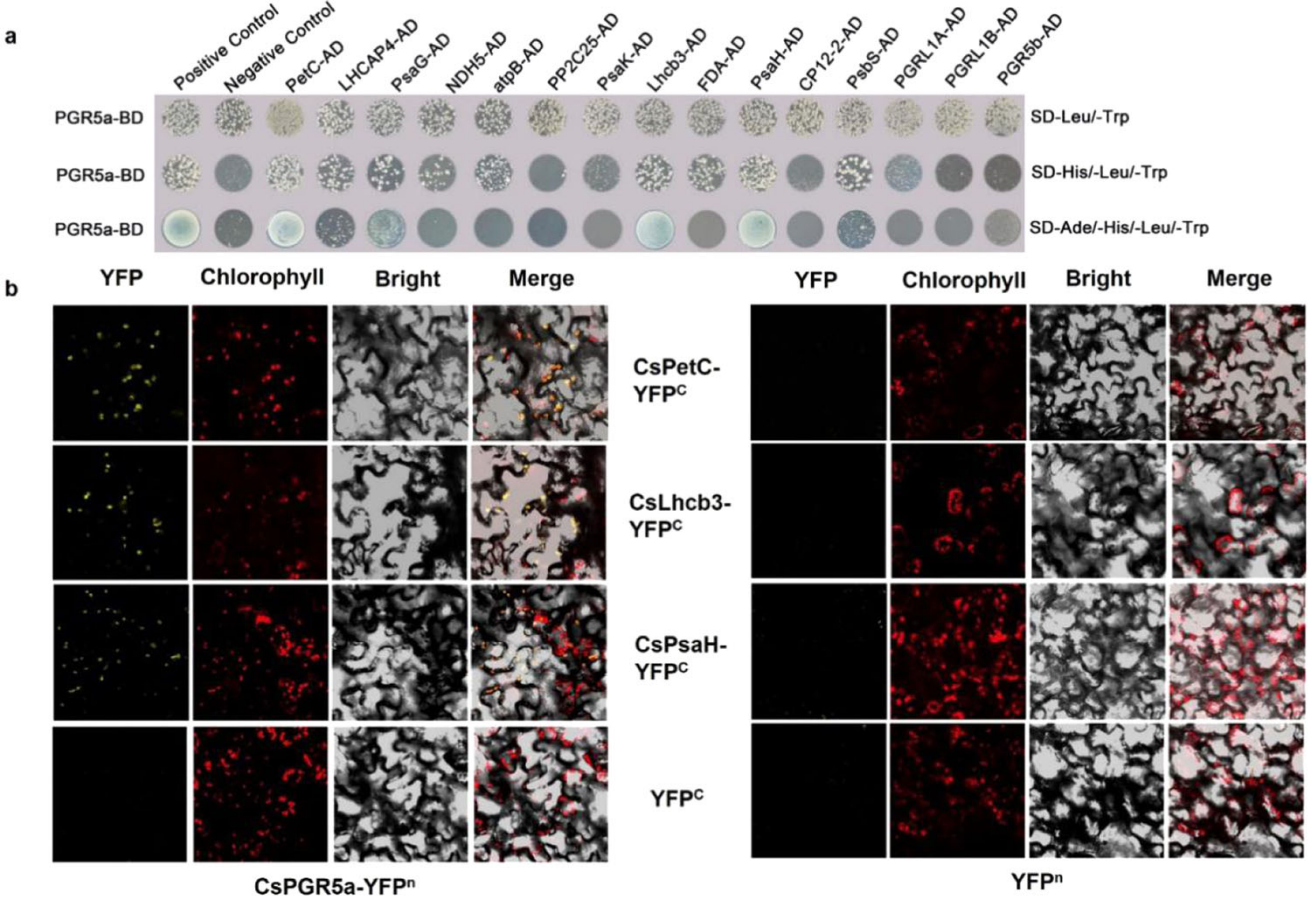
Analysis of the interactions between CsPGR5a and candidate proteins. **a** Yeast two-hybrid assay analysis. CsPGR5a was fused to the DNA-binding domain (PGR5a-BD), while the candidate proteins were fused to the activation domain (PetC-AD, LHCAP4-AD, PsaH-AD, NDH5-AD, atpB-AD, PP2C25-AD, PsaK-AD, Lhcb3-AD, FDA-AD, PsaG-AD, CP12-2-AD, PsbS-AD, PGRL1A-AD, PGRL1B-AD, and PGR5b-AD). pGBKT7-53+pGADT7-T and pGBKT7-Lam + pGADT7-T were used as positive and negative controls, respectively. **b** BiFC assay of the interactions of PetC, Lhcb3, and PsaH with PGR5a. Plasmids encoding fusion constructs with the N- or C-terminal parts of YFP (PGR5a-YFP^n^, PetC-YFP^c^, Lhcb3-YFP^c^, and PsaH-YFP^c^) were separately transiently expressed in *Nicotiana benthamiana* leaves. The yellow signals indicate YFP fluorescence; the magenta signals indicate chloroplast autofluorescence

To further analyze the relationships between PGR5 and other electron transporters, we first used a construct in which CsPGR5a was fused to the DNA-binding domain (PGR5a-BD) as bait to screen positive clones in the cucumber cDNA library through yeast two-hybrid assays. Twelve proteins that appeared more than three times were selected as candidate interacting proteins: PetC, LHCAP4, PsaH, PnsL5, atpB, PP2C25, PsaK, Lhcb3, FDA, PsaG, CP12-2, and PsbS. Unexpectedly, the known PGR5-interacting protein PGRL1 was not detected in this screening; however, we still constructed PGRL1A, PGRL1B, and PGR5b plasmids fused to activation domains (ADs; PGRL1A-AD, PGRL1B-AD, and PGR5b-AD, respectively) for subsequent experiments (Table S3). The results of the yeast two-hybrid assays (Fig. 2a) showed that PGR5a interacted with Lhcb3, PetC, PsaH, FDA, PsaH, atpB, PsbS, and LHCAP4 on SD/-Leu/-Trp/-His plates, but there were only three positive clones on SD/-Leu/-Trp/-Ade/-His plates; therefore, we considered FDA, PsaG, atpB, LHCAP4 and PsbS to interact weakly with PGR5a. The positive interactions between PGR5a and the three candidates (PetC, Lhcb3, and PsaH) were also confirmed via BiFC assays (Fig. 2b).

The mature intact PetC protein of the cyt *b*_6_*f* complex of spinach, cucumber, and Arabidopsis chloroplasts comprises 179, 178, and 179 residues, respectively, and is highly conserved (Fig. S5a)^13^. Like PetC in other species, CsPetC had a predicted transmembrane domain in the 67~86 residue site (Fig. S5b), an N-terminal stromal part, and a C-terminal soluble domain on the positive side of the membrane (Fig. S5c)^14^. Therefore, we divided the mature CsPetC into three parts: a (i) stromal part (Str, 50~66); (ii) transmembrane part (TM, 67~86); and (iii) lumenal part (Lum, 87~227) (Fig. S5c). The results of the yeast two-hybrid assay and BiFC assay analysis showed that CsPGR5 interacted with the lumenal part of CsPetC (Fig. S6).

**Fig. 3:**
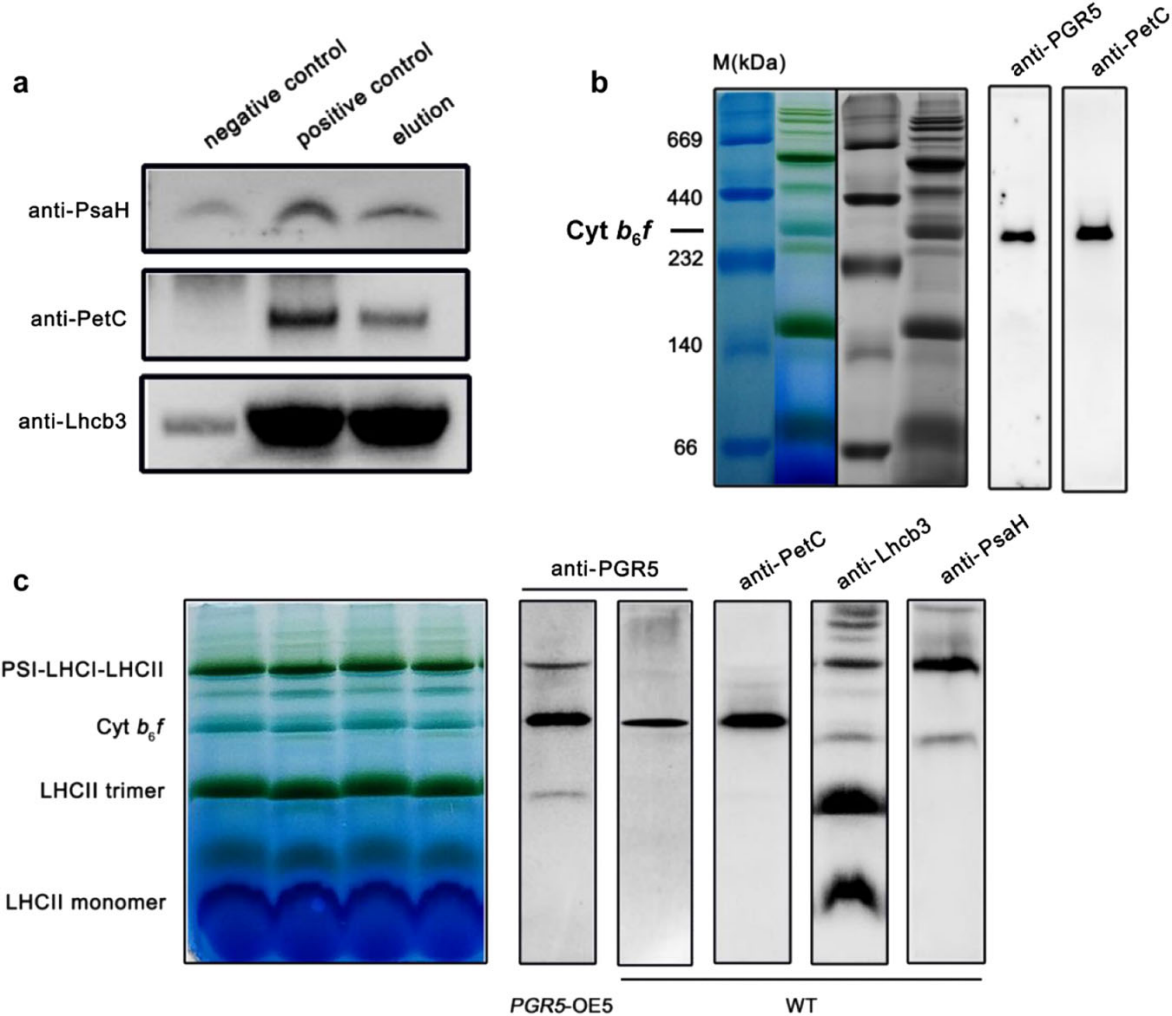
Coimmunoprecipitation assay analysis of PGR5a and separation of thylakoid membrane protein complexes by blue native PAGE followed by immunoblotting. **a***CsPGR5a-HA*, *CsPetC*, *CsLhcb3*, and *CsPsaH* were coexpressed in *N. benthamiana*, and CoIP was performed by using an anti-HA antibody. Tobacco thylakoid membranes coexpressing empty vectors together with *HA*, *CsPetC*, *CsLhcb3*, and *CsPsaH* were used as negative controls, and purified thylakoid membranes (Input) were used as positive controls. Western blotting was performed by using anti-PsaH, anti-PetC, and anti-Lhcb3 antibodies. **b** Immunoblotting analysis of cucumber thylakoid membrane protein complexes by using anti-PGR5 and anti-PetC antibodies. **c** BN-PAGE of tobacco thylakoid membrane proteins with or without overexpression of *CsPGR5a*, anti-PGR5, anti-PetC, anti-Lhcb3, and anti-PsaH antibodies was used for immunoblotting

Second, a coimmunoprecipitation (CoIP) assay was carried out. When cucumber thylakoid membranes were purified and incubated with a PGR5-specific antibody, PetC, PsaH and Lhcb3 were identified via MS (Table S4), and the identified amino acid sequences of CsPetC (DAFGNDVFADEWLK and GDPTYLVVEK) were detected in the lumenal part of CsPetC. In addition, the other subunits of PSI, LHCI, and cyt *b*_6_*f* were also identified; however, PGRL1 was still undetected. Because the PGR5 antibody could not recognize PGR5a or PGR5b among the total PGR5 proteins, we used *Agrobacterium tumefaciens* strain EHA105 to transiently transform *N. benthamiana* leaves with HA-conjugated CsPGR5a (CsPGR5a-HA), CsPetC, CsLhcb3, and CsPsaH. Western blot analysis of the PGR5-HA coimmunoprecipitates confirmed that PGR5a interacted with PetC, Lhcb3, and PsaH (Fig. 3a).

Finally, we separated the thylakoid membrane protein complexes by blue native PAGE, a method for analyzing protein-protein interactions^15^, followed by immunoblotting. To show the specificity of the PGR5 antibody for PGR5 in membrane protein complexes, different tobacco lines (WT, *pgr5#101*, *pgr5#21*, and *pgr5ab*) were used for testing. Fig. S7 shows that when *pgr5a* was knocked out (*pgr5#101*, *pgr5#21*), a weak blot was detected in the cyt *b*_6_*f* complex band because of the presence of PGR5b. When both *pgr5a* and *pgr5b* were knocked out (*pgr5ab*), there was no PGR5 signal in the immunoblot, indicating that the anti-PGR5 antibody is specific for the PGR5 protein. When cucumber thylakoid membranes were used, as expected, PGR5 was detected in the cyt *b*_6_*f* complex band. However, we failed to detect the PGR5 signal in PSI and LHCII supercomplexes, which may have been due to the low abundance of this protein^7^ or because the processes of extraction and separation were not gentle enough, resulting in the dissociation of PGR5 from the other supercomplexes. The results also revealed strong binding of PGR5 to the cyt *b*_6_*f* complex (Fig. 3b). Therefore, we overexpressed *CsPGR5a* in common tobacco to increase PGR5 abundance in other supercomplexes. Fortunately, we detected two faint PGR5 signals in the PSI-LHCI-LHCII and LHCII trimer bands, which provided evidence that PGR5a can attach to PSI and LHCII (Fig. 3c).

### PGR5-dependent CEF is partially related to the LHCII state transition

**Fig. 4:**
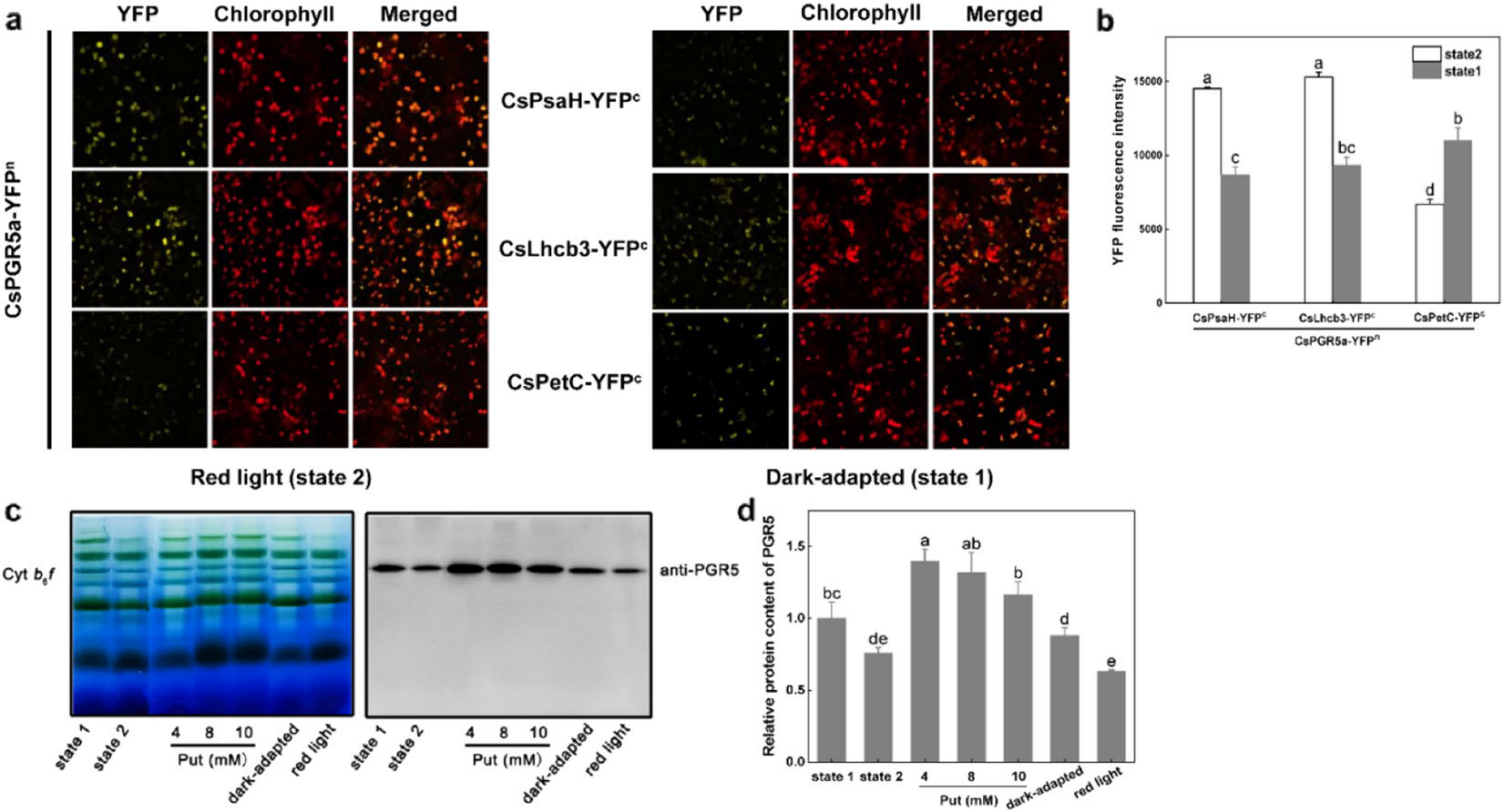
Effects of state transitions and exogenous Put on the association state of PGR5. **a** The BiFC signal of the PGR5a interaction with PsaH, Lhcb3 and PetC was detected after transient expression in tobacco leaves. *N. benthamiana* plants with the same growth conditions were selected, and leaves at the same position were injected with the same combination of *A. tumefaciens*. The seedlings were adapted for 3 days followed by red light illumination and dark adaptation for 30 min, after which the YFP signal was detected. **b** Quantification of YFP fluorescence intensity. **c** BN-PAGE with immunoblotting of thylakoid membrane complexes. To arrest the thylakoid membranes in state 1, cucumber discs were incubated with the kinase inhibitor staurosporine at 100 nM for 80 min and then in 20 μM DCMU for 10 min; to isolate state 2-arrested thylakoid membranes, cucumber discs were incubated with the phosphatase inhibitor NaF at 100 mM for 50 min and then in 5 μM carbonyl cyanide p-(trifluoromethoxy)phenylhydrazone (Sigma) for 10 min. Exogenous Put at concentrations of 4, 8, and 10 M was incubated together with the cucumber leaf discs for 60min. The total bands separated by BN-PAGE were used as a loading control. **d** Relative protein content of PGR5. The different letters indicate significant differences between treatments (*P* < 0.05) according to Tukey’s test

The relationship between CEF and the LHCII state transition has been controversial for a long time. To better understand this relationship, we induced state transitions by light or chemical induction, and the thylakoid membranes were then purified and separated by sucrose gradient ultracentrifugation into PSII (band I)- and PSI (band II)-complexes (Fig. S9a), which were subsequently analyzed by SDS-PAGE (Fig. S9b). The results of immunoblotting using anti-Lhcb1,2,3 antibody showed that the abundance of LHCII in the PSI complex was higher in state 2 (or red light) than in state 1 (or dark adapted), proving the efficiency of the treatments for state transition (Fig. S9c). The transiently transformed *N. benthamiana* plants were then dark adapted (state 1) and exposed to red light (state 2) for 30 min on the third day, and the strength of the YFP signal was measured as an indicator of the interaction intensity. Figure 4a and b shows that the YFP fluorescence intensity of the interaction of PGR5a with PsaH and Lhcb3 was 67 and 64% stronger in state 2 than in state 1, respectively. In contrast, the interaction of PGR5a with PetC was 64% stronger in state 1 than in state 2. Furthermore, we arrested the cucumber thylakoid membranes in either state 1 or state 2 and isolated them for BN-PAGE and immunoblot analysis. To arrest the membranes in state 1, cucumber leaf discs were incubated with 100 nM staurosporine for 80 min followed by 20 μM 3-(3,4-dichlorophenyl)-1,1-dimethylurea (DCMU) for 10 min. To obtain state 2-arrested thylakoid membranes, leaf discs were treated with 100 mM sodium fluoride (NaF) for 50 min and with 2.5 μM carbonyl cyanide p-trifluoromethoxyphenylhydrazone (FCCP) for 10 min. Dark-adapted and red light-illuminated leaf discs were also used. The results showed that the PGR5 abundance in the cyt *b*_6_*f* complex was ~40% higher in state 1 than in state 2 (Fig. 4c, d), which revealed that PGR5 dissociated from cyt *b*_6_*f* when LHCII transitioned from state 1 to state 2. Overall, when the state transition did not occur, most PGR5a associated with cyt *b*_6_*f* in state 1, while a large number of PGR5a molecules attached to PSI in state 2.

### PGR5a has the same function in cucumber and tobacco

**Fig. 5:**
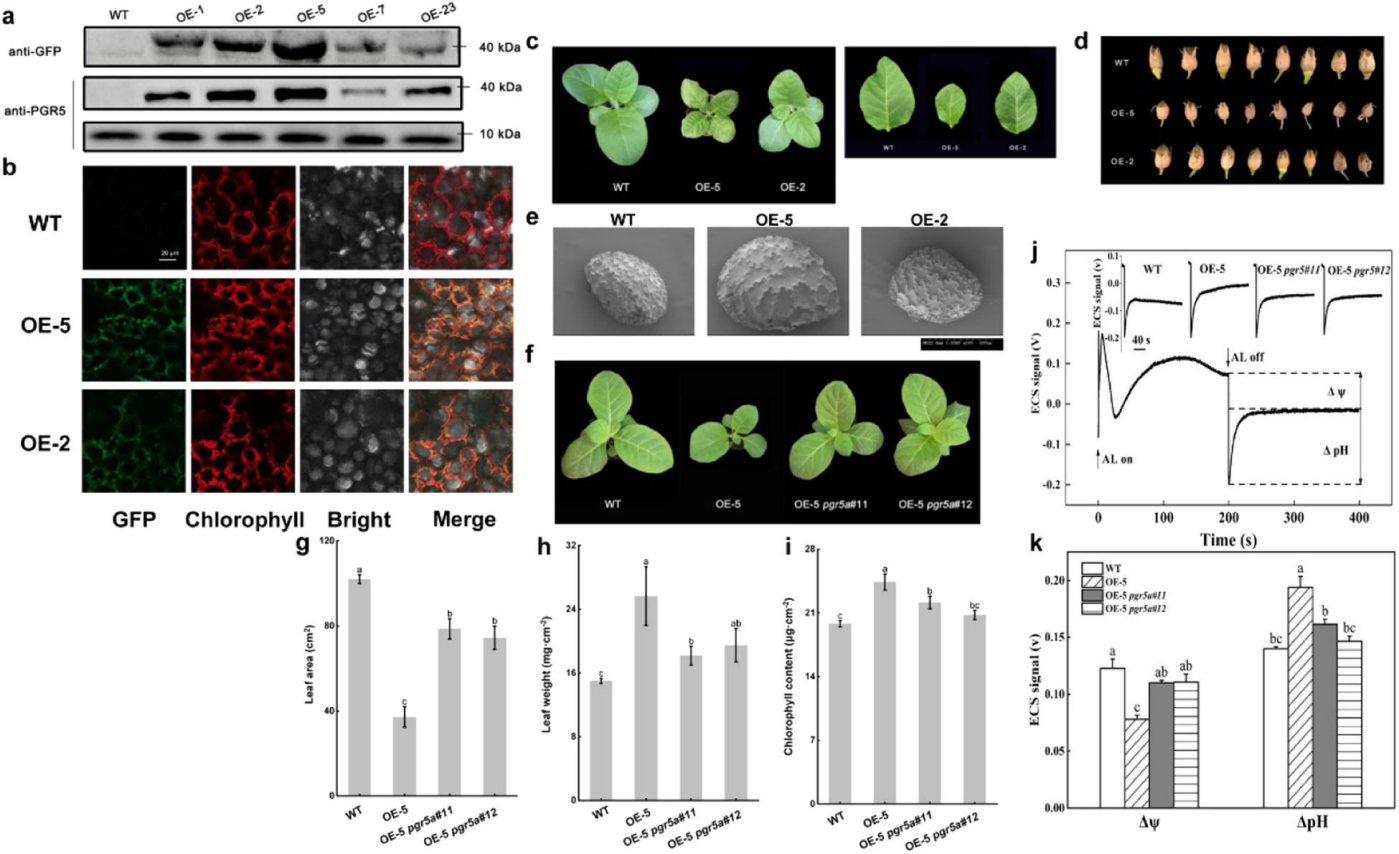
Overexpression of *CsPGR5a* in common tobacco. **a** Western blot of the WT and different transgenic lines (OE-5 and OE-2) using anti-GFP and anti-PGR5 antibodies. **b** GFP signal of tobacco leaves in the WT and different transgenic lines. **c**–**e** Phenotypes of tobacco seedlings, leaves, capsules, and seeds of the WT and different transgenic lines. The images of the seeds were taken by an S3000 scanning electron microscope (Hitachi, Tokyo, Japan). **f** Tobacco *PGR5a* was knocked out in the OE-5 line, and the phenotypes of OE-5 *pgr5#11* and OE-5 *pgr5#11* recovered to a phenotype similar to that of the WT. **g**–**i** Leaf area, leaf weight per area, and Chl content per area of WT, OE-5, OE-5 *pgr5#11*, and OE-5 *pgr5#11*. **j**, **k** Two *pmf* components of WT, OE-5, OE-5 *pgr5#11*, and OE-5 *pgr5#11*. The different letters indicate significant differences between treatments (*P* < 0.05) according to Tukey’s test

We overexpressed cucumber *PGR5a* via *GFP* in common tobacco (NC89) and selected two transgenic lines with different expression levels (the one with higher expression was named OE-5, and the one with lower expression was named OE-2; Fig. 5a, b). The phenotypes of the overexpression lines differed from the phenotype of the wild-type (WT) line; this was especially true for OE-5, which grew to a smaller size than the WT and had smaller but thicker leaves (Fig. 5c). The capsules of OE-5 were smaller than those of OE-2 and the WT; however, the seeds of OE-5 were larger than those of OE-2 and the WT (Fig. 5d, e). To eliminate the possibility that these differences were caused by overaccumulation of exogenous PGR5a or GFP, we knocked out the endogenous tobacco *PGR5a*-encoding gene in the *CsPGR5a*-overexpressing line OE-5. As shown in Fig. 5f–i, the phenotypes of OE-5 *pgr5a#11* and OE-5 *pgr5a#12* were similar to the phenotype of the WT. Furthermore, the leaf area, leaf weight per area, and chlorophyll content per area of the two knockout lines were similar to those of the WT, which indicated that the change in phenotypes was caused by *PGR5a*. If CsPGR5a functions in tobacco, it should increase the proton gradient across the thylakoid membrane; thus, the two components of the *pmf* were detected in the four transgenic lines. The results showed that OE-5 had the highest ΔpH and the lowest Δψ, but when *NtPGR5a* was knocked out in OE-5, the ΔpH and Δψ were similar to those of the WT (Fig. 5j, k). Taken together, these results indicated that PGR5a had the same characteristics and function in both cucumber and tobacco; hence, we deemed the tobacco transgenic lines suitable for subsequent experiments.

**Fig. 6:**
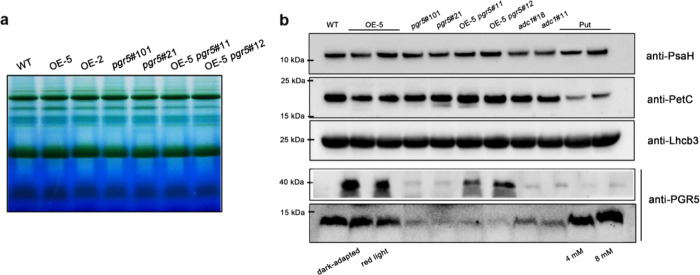
BN-PAGE and western blotting of tobacco thylakoid membrane proteins. The *pgr5* overexpression and deficient lines were developed in the background of common tobacco NC89. The *PGR5a* coding sequence was cloned into cucumber cDNA. To explain whether *PGR5a* in cucumber and tobacco has the same function, we knocked out PGR5a in tobacco in the CsPGR5 overexpression line. **a** BN-PAGE analysis of the thylakoid membrane complexes of WT, OE-5, OE-2, *pgr5#101*, *pgr5#21*, OE-5 *pgr5#11*, and OE-5 *pgr5#11*. **b** Western blotting analysis of complexes of WT, OE-5, OE-2, *pgr5#101*, *pgr5#21*, OE-5 *pgr5#11*, and OE-5 *pgr5#11* by using anti-PGR5, anti-Lhcb3, anti-PetC, and anti-PsaH antibodies

### PGR5a participates in proton transport at the cyt *b*_6_*f* complex, and its assembly, but not its content, changed with state transition

We generated *pgr5#101* and *pgr5#21* tobacco *PGR5a*-knockout lines. Figure 6a shows that regardless of whether PGR5 was overexpressed or knocked out, the integrity of thylakoid membrane protein complexes was not altered. As shown in Fig. 6b, the dim PGR5 signal in the western blots revealed that PGR5b could not compensate for functionally deficient PGR5a. By using an anti-PGR5 antibody, we detected two PGR5-including bands in the *CsPGR5a*-overexpressing lines of tobacco created by fusing GFP to the C-terminus of CsPGR5a. We found that the content of PGR5 did not differ between dark-adapted (state 1) and red light-illuminated plants (state 2); thus, state transition affected the assembly but not the content of PGR5. In addition, the abundance of Lhcb3 did not differ among the state 1, state 2, PGR5 overexpression and PGR5 knockout conditions (Fig. 6b). Interestingly, overexpression of *CsPGR5a* in tobacco decreased the level of PetC, so we hypothesized that PGR5 in cyt *b*_6_*f* may affect the function of PetC (Fig. 6b).

We have demonstrated that Put increases PGR5-dependent CEF by regulating the pH in the lumen^16^. It is well known that PGR5 participates in proton gradient regulation, which can increase the ΔpH across the thylakoid membrane. In contrast, Put in the lumen can decrease the ΔpH^16,17^. In this experiment, we incubated cucumber leaf discs together with Put under a vacuum system, and BN-PAGE and immunoblot analysis showed that the abundance of PGR5 increased significantly at the cyt *b*_6_*f* site (Fig. 4b). Accordingly, exogenous Put also increased the total PGR5 levels in the thylakoids (Fig. 6b), indicating that the decreased ΔpH greatly enhanced PGR5 abundance and association with cyt *b*_6_*f* to enable transport of protons from the outside environment into the lumen. The theoretical transmembrane domain in the N-terminus of PGR5a could also provide evidence for its proton transport character (Fig. S3c).

## Discussion

In this work, we identified two PGR5s in cucumber, as most studies have been based on Arabidopsis and *C. reinhardtii*, each of which has only one PGR5. Interestingly, in general, the two PGR5s had characteristics similar to those of PGR5s in some algae and plants and exhibited positive responses to changing environments. The plant materials used in this work were cucumber and common tobacco, both of which have two different PGR5s. The mature parts of the two PGR5 amino acid sequences within each species had high levels of identity: CsPGR5a and CsPGR5b shared 89.04% identity, and NtPGR5a and NtPGR5b shared 98.63% identity (data not shown). However, we did not find interactions between the two PGR5s (Fig. 2). As in cucumber, the expression level of PGR5a in tobacco was significantly higher than that of PGR5b (Fig. S8); thus, PGR5a, rather than PGR5b, was determined to play the dominant role in CEF. However, the question remains as to why some photosynthetic organisms have evolved two different but similar PGR5s. In the tobacco *pgr5a*-knockout mutant, the abundance of total PGR5 protein was extremely low (Fig. 6b), implying that PGR5b is incapable of compensating for defective PGR5a. Furthermore, the absence of PGR5a may impair the stability of PGR5b.

In Arabidopsis, PGR5 and PGRL1 are thought to interact both functionally and physically, forming a heterodimer that shuttles electrons from PSI via Fd to cyt *b*_6_*f*^6,7^. Unexpectedly, we failed to detect an interaction between PGR5 and PGRL1 via yeast two-hybrid assay screening of a cucumber cDNA library or via CoIP assays using cucumber thylakoid membranes incubated together with an anti-PGR5 antibody. Thus, the existence of a relationship between PGR5 and PGRL1 in species other than Arabidopsis and *C. reinhardtii* should be reconsidered. PGR5 is present in all photosynthetic organisms, whereas PGRL1 is specific to green algae and plants^6^. Recently, Dann and Leister^18^ found a PGRL1-like protein in the cyanobacterium *Synechocystis*. AtPGRL1-SynPGR5 is not functional, while AtPGR5-SynPGRL1-LIKE exhibits CEF activity in cyanobacteria, implying the high conservation of sequence and function in PGR5 and the irreplaceable role of PGR5 in CEF.

PGR5 is a small protein that accumulates to low levels and lacks a metal-binding motif; therefore, it is unlikely that PGR5 itself directly transports electrons from Fd to PQ^12^. Given that FNR binds to the cyt *b*_6_*f* complex with a specific stoichiometry in purified spinach^19,20^, it has been proposed that the cyt *b*_6_*f* complex acts as an Fd:PQ reductase in CEF and that a PGR5-related complex functions in this process^21^. Interestingly, a recent publication by Buchert et al.^22^ concluded that PGR5 was required for efficient Q cycling within the cyt *b*_6_*f* complex, which provided a new model for PGR5-CEF. In our experiments, a high abundance of PGR5 was detected in the cyt *b*_6_*f* complex, supporting the interdependence of PGR5 and the cyt *b*_6_*f* complex. Not only electron transfer but also proton transit across the thylakoid membrane takes place at cyt *b*_6_*f*; thus, we hypothesized that PGR5 may participate in proton gradient regulation when it is attached to cyt *b*_6_*f*. We incubated leaf discs together with exogenous Put, which modulates the *pmf* in thylakoids by dissipating the ΔpH and favoring Δψ^16,17,23^. The amount of PGR5 at cyt *b*_6_*f* complex sites significantly increased, and the total PGR5 content also increased slightly (Figs. 4b and 6b). Furthermore, the PetC level decreased when *CsPGR5* was overexpressed in tobacco (Fig. 6b). PetC is the Rieske [2Fe-2S] protein subunit of the cyt *b*_6_*f* complex involved in proton translocation into the thylakoid lumen, and a specific alteration within its amino acid sequence makes the cyt *b*_6_*f* complex hypersensitive to lumenal acidification. A mutant resulting from this alteration was named *proton gradient regulation 1* (*pgr1*) because the Arabidopsis *pgr1* mutant makes it difficult for a high ΔpH to be established across the thylakoid membrane to induce thermal energy dissipation^24,25^, implying that PetC and PGR5 have similar functions to some degree and that PGR5 can partially replace PetC to play a role in proton gradient regulation. We detected a single PetC signal in cyt *b*_6_*f*, proving that PGR5 interacted with PetC at the site of the cyt *b*_*6*_*f* complex. PetC in the cyt *b*_6_*f* complex functions as the primary oxidant of plastoquinol on the lumenal side of the membrane and as an electron donor for the cytochrome *f* subunit and may have a dynamic role in catalyzing proton transfer at the membrane interface^13^. PGR5 interacts with the lumenal part of PetC, which also indicates an electron and proton transfer regulatory role of PGR5 in cyt *b*_6_*f*. Lhcb3 is one of the subunits of the M-LHCII trimer (the other two are Lhcb1 and Lhcb2), which is a component of PSII; therefore, the interaction between PGR5 and Lhcb3 is probably associated with state transition. However, unlike Lhcb1 and Lhcb2, Lhcb3 lacks the N-terminal phosphorylation site (Thr/Ser residue) involved in the state transition; moreover, Arabidopsis *Lhcb3*-knockout plants have higher rates of transition from state 1 to state 2 than WT plants do because the M-LHCII trimer has three potential phosphorylation sites in Lhcb3-knockout plants, while only two such sites are present in WT plants^26,27^. From this perspective, Lhcb3 seems to protect PSI by slowing the rate of state transition. Interestingly, PsaH is the docking site for the LHCII trimer on PSI^28^. Compared with WT Arabidopsis, PGR5-overexpressing lines of Arabidopsis exhibit higher phosphorylation levels of LHCII in the dark^29^. All of the above findings provide reasonable evidence that a relationship between the state transition and PGR5-dependent CEF exists. Accordingly, in this study, the interaction strength between Lhcb3 (as well as PsaH) and PGR5 was much stronger in state 2 than in state 1, while the interaction strength between PetC and PGR5 exhibited the opposite pattern (Fig. 4a). Furthermore, the abundance of PGR5 at the cyt *b*_*6*_*f* complex site decreased upon the transition from state 1 to state 2 (Fig. 4b). Even so, we consider that state transition can trigger CEF, but it is not indispensable for CEF, since both state transition and CEF can be driven independently^10^. In conclusion, we propose that PGR5 is a small mobile protein that functions when attached to protein complexes and that neither excess PGR5 nor the absence of PGR5 alters the structures of these complexes (Fig. 6a)^29^. The function of PGR5 is partly influenced by the LHCII state transition. These novel findings raise new questions needing further study. (1) Like cucumber and tobacco, other species of plants and algae have evolved second PGR5s; what are the structural and functional roles of these molecules? (2) What is the exact relationship between PGR5 and PGRL1? Is there a connecting factor for the generation of a PGR5-PGRL1 heterodimer? (3) Does a PSI-LHCII-cyt *b*_*6*_*f* supercomplex with PGR5 exist in higher plants? (4) Many more thylakoid proteins can undoubtedly interact with PGR5; what are they, and what functions are associated with PGR5? (5) Given that state transition and other factors, such as the redox state of the thylakoid membrane, can drive the migration of PGR5, what are the exact mechanisms of PGR5 dissociation and assembly in supercomplexes? (6) PGR5 may partially replace PetC to participate in proton gradient regulation in cyt *b*_*6*_*f* complexes, but how might this work mechanically?

## Materials and methods

### Identification and sequence analysis of CsPGR5

The *PGR5* sequences of cucumber in the Cucurbit Genomics Database (http://cucurbitgenomics.org/) were queried, and we then compared the results with the content within the NCBI database. The amino acid sequences of PGR5 proteins in 10 species were aligned using the European Bioinformatics Institute tool (https://www.ebi.ac.uk/Tools/sss/ncbiblast/), and the conservation values were calculated using Jalview (http://www.jalview.org/). In addition, the molecular weight and theoretical isoelectric point were calculated with the ProtParam tool (https://web.expasy.org/protparam/). The phosphorylation sites were predicted online (http://www.cbs.dtu.dk/services/NetPhos/), the transmembrane regions were predicted with TMpred (https://embnet.vital-it.ch/software/TMPRED_form.html), and the 3D structures were predicted using SWISS-MODEL (https://swissmodel.expasy.org). A phylogenetic tree was constructed using MEGA (version 7.0), with the maximum likelihood method and bootstrap analysis (1000 replicates). In addition, the 2000 bp genomic sequences upstream of the initiation codons of the two cucumber PGR5 genes were obtained with TBtools and used for putative *cis*-element prediction via PlantCARE (http://bioinformatics.psb.ugent.be/webtools/plantcare/html/).

### Plant materials and treatments

Cucumber (*C. sativus* L.) seedlings of inbred line 9930 were used in this work. When the third leaf had fully expanded, the seedlings were subjected to one of the following treatments: exogenous ABA (100 μM), SA (100 μM), MeJA (100 μM), or Put (8 mM) spraying of the leaves; 20% PEG 6000 (to mimic drought stress); 4 °C (to induce cold stress); or 100 mM NaCl (to induce salt stress). The second leaves were collected at 0, 1, 2, 6, 12, 24, 48, and 72 h. The different light intensities were set at 0, 80, 185, 280, 460, 700, 925, and 1250 μmol photons m^−2^ s^−1^.

For tobacco transformation, the common tobacco line NC89 (*N. tabacum* L.) was used as the WT. Transformation of tobacco was performed using a leaf disc cultivation method described previously^30^. CsPGR5a-overexpressing lines were identified via genomic PCR, qRT-PCR, western blotting using an anti-GFP monoclonal antibody, and GFP fluorescence analysis. The CRISPR/Cas9 genome-targeting system was used to generate *pgr5*-knockout mutants according to previously described methods^31^, and the mutants were identified by sequencing analysis (Fig. S10).

### Gene expression analysis

Total RNA was isolated with the RNAsimple Total RNA Kit (Tiangen, DP419) following the protocols of the manufacturer. The RNA concentration was measured using a NanoDrop spectrophotometer (Thermo Scientific, USA). cDNA synthesis and real-time PCR were performed according to the methods of He et al.^30^. *Actin* from cucumber (locus name: XM_011659465) or common tobacco (locus name: XM_016658252) was used as a reference gene.

### Yeast two-hybrid assays

A yeast two-hybrid experiment was performed as described by Guan et al.^32^. PGR5a-BD was used as bait to screen for positive clones with a cucumber cDNA library. The selected candidate proteins (Table S3) were fused to ADs to construct prey, and CsPGR5a was fused to BD to construct bait. The prey and bait plasmids were used to cotransform the yeast strain Y2H Gold, and the transformed yeast was plated onto SD-Leu/-Trp, SD-His/-Leu/-Trp, and SD-Ade/-His/-Leu/-Trp plates. Yeast cells cotransformed together with pGBKT7-53 and pGADT7-T were used as positive controls, and yeast cells transformed with pGBKT7-Lam and pGADT7-T were used as negative controls.

### BiFC assays

BiFC assays were performed essentially as described by Jiang et al.^33^. The full-length CDSs (without the stop codons) of PGR5a and the selected PGR5a-interacting proteins from yeast two-hybrid assays were amplified, cloned, and inserted into truncated eYFP fusion vectors. After transformation of the A. tumefaciens strain EHA105, the indicated EHA105 combinations were used for transient transformation of *N. benthamiana* leaves. YFP fluorescence was visualized on the third day after transformation with an LSM 780 confocal microscope (Zeiss, Germany).

### Isolation of thylakoid membranes

Thylakoids were isolated from fresh leaves ground in a precooled grinding buffer (50 mM HEPES/KOH [pH 7.8], 330 mM sorbitol, 2 mM EDTA-Na_2_, 2 mM MgCl_2_·6H_2_O, 10 mM NaHCO_3_, 0.05% BSA, 5 mM ascorbic acid with or without 10 mM sodium fluoride). The suspension was subsequently filtered and centrifuged at 4000 × *g* at 4 °C for 4 min. The pellet was resuspended in shock buffer (50 mM HEPES/KOH [pH 7.6], 2 mM MgCl_2_·6H_2_O, 1 mM EDTA-Na_2_ with inhibitor cocktail) and incubated on ice for 30 min to lyse the intact chloroplasts; the mixture was then centrifuged at 12,000 × *g* at 4 °C for 4 min. The pellet was subsequently washed with wash buffer (50 mM BisTris/HCl [pH 7.0], 330 mM sorbitol) 1~2 times. Finally, the thylakoid concentration was adjusted to 1 mg Chl ml^−1^ with sample buffer (25 mM BisTris/HCl [pH 7.0], 20% [v/v] glycerol).

### Separation of thylakoid membrane protein complexes by BN-PAGE

Purified thylakoid membranes were dissolved in an equal volume of 2% DM and incubated for 20 min on ice. The insoluble fraction was removed by centrifugation at 12,000 × *g* at 4 °C for 10 min. The supernatant was then supplemented with a one-tenth volume of Serva Blue G buffer (100 mM BisTris/HCl [pH 7.0], 500 mM 6-amino-n-caproic acid, 5% [w/v] Serva Blue G, 30% [w/v] glycerol). For BN-PAGE, there was an acrylamide gradient of 3.5–12% (30% T, 3.2% C) in the separation gel and 4% (30% T, 20% C) in the stacking gel. Electrophoresis was performed at 4 °C, with a gradual increase in voltage as follows: 75 V for 30 min, 100 V for 30 min, 125 V for 30 min, and 150 V until the sample reached the end of the gel (however, if the gel was to be used for immunoblotting in the next step, electrophoresis was stopped when the sample had migrated two-thirds of the way to the end of the gel).

### Immunoblotting after BN-PAGE

Before being transferred to a PVDF membrane, each BN-PAGE gel was equilibrated in Tris-glycine/MeOH transfer buffer (25 mM Tris, 192 mM glycine, 20% [v/v] methanol with 0.037% SDS) under gentle shaking for 30 min at room temperature (RT). Subsequent transfer to the PVDF membrane was then conducted as normal, except that the transfer time was prolonged to 3 h. After transfer, the PVDF membrane was rinsed in 100% MeOH to remove the Serva Blue G. The membrane was blocked for 1 h in 5% skim milk at RT, after which it and commercially obtained primary antibodies (PhytoAB, USA) were incubated at 4 °C overnight. After incubation with a secondary antibody (PhytoAB, USA) for 1 h at RT, the membranes were assessed by the use of a ChemiDoc Touch (Bio-Rad, USA) system.

### CoIP analysis

A CoIP assay was performed as described by Hertle et al.^7^, with modifications. For CoIP of the native PGR5 protein, a rabbit antibody specific for PGR5 was affinity purified and raised against synthetic peptides with the sequence IRLAKKNGERLGFLA (which is exactly the same as that of AtPGR5, CsPGR5a/b and NtPGR5a/b), and thylakoids (at a final concentration of 1 mg Chl/ml) together with a PGR5-specific antibody were incubated together overnight in TBS at a final concentration of 50 μg ml^−1^. The antibody-treated thylakoids were solubilized in TBS consisting of 1.5% (w/v) DM, and the sample was incubated for 30 min. After centrifugation (10 min, 12,000 × *g*, 4 °C), the supernatant was collected, supplemented with 50 µl of Protein A-conjugated magnetic beads (MCE, China) (which had been preequilibrated with TBS supplemented with 0.08% [w/v] DM), and shaken for 2 h at 4 °C. The protein-antibody-magnetic bead complexes were washed three times with 10 volumes of TBS consisting of 0.08% (w/v) DM. The bound proteins were subsequently eluted with 2× Laemmli buffer at 45 °C for 1 h and then analyzed via SDS-PAGE and MS.

For CoIP of the HA-PGR5 protein, the *A. tumefaciens* strain EHA105 was used to transiently transform *N. benthamiana* leaves with CsPGR5a-HA, CsPetC, CsLhcb3, and CsPsaH. The thylakoid membranes were purified after 3 days and solubilized in TBS consisting of 1.5% (w/v) DM at a final concentration of 1 mg Chl·ml^−1^ for 30 min. After centrifugation at 12,000 *×* *g*, the supernatant and 70 μl of anti-HA magnetic beads (MCE, China) (which had been preequilibrated with TBS consisting of 0.08% [w/v] DM) were incubated together and shaken overnight at 4 °C. The following steps were the same as described above, and a protease inhibitor cocktail was added to all the buffers. The eluate was analyzed via western blotting.

### Western blotting

Tricine-SDS-PAGE^34^ was preferentially used for the separation of PGR5 and PsaH (<15 kDa). Five micrograms of Chl from thylakoid membranes was loaded onto a 4% stacking gel (49.5% T, 3% C) after it was prerun at 30 V for 10 min. We used an appropriate separating gel of 16% acrylamide consisting of 6 M urea (49.5% T, 3% C). The electrophoresis buffer consisted of 1× anode buffer (0.1 M Tris/HCl [pH 8.9]) and 1× cathode buffer (0.1 M Tris/HCl [pH 8.25], 0.1 M Tricine, 0.1% [w/v] SDS). Electrophoresis was initially performed at 30 V for the stacking gel, and a voltage of 120 V was maintained for the separation gel. SDS-urea-PAGE was used to separate PetC and Lhcb3. The stacking and separation gels were set at 4% and 15% with 6 M (30% T, 3.3% C), respectively. Ten micrograms of Chl from the thylakoid membranes was loaded in the stacking gel, which was then run at 80 V; a voltage of 140 V was then maintained until the proteins ran to the end of the gel.

After electrophoresis, the proteins on the gels were transferred to a 0.45 μm PVDF membrane for 30 min at 0.05 mA cm^−2^. The remaining steps were the same as described in “Immunoblotting after BN-PAGE”.

### Induction of state 1 and state 2

We used two methods to induce state 1 and state 2. In the first methods, plants or leaf discs were maintained in the dark (induction of state 1) or exposed to red light (induction of state 2) for 30 min. The second method was performed according to a protocol described by Takahachi et al.^35^, with modifications. To arrest the thylakoid membranes in state 1, cucumber leaf discs were incubated in 100 nM staurosporine for 80 min to inhibit phosphorylation of LHCII proteins and then in 20 μM DCMU for 10 min to inhibit the reduction of Q_B_ at PSII so that the PQ pool would be oxidized in the light. To isolate state 2-arrested thylakoid membranes, we treated leaf discs with the phosphatase inhibitor NaF at 100 mM for 50 min followed by 2.5 μM FCCP, an uncoupling agent that causes state 2 transitions via depletion of ATP, for 10 min.

### Isolation of PSII and PSI complexes

One milligram of purified thylakoid membranes was solubilized in 1% DM to a final concentration of 1 mg/ml, and the sample was loaded on a 10.5 ml 0~1.0 M SDG (consisting of 20 mM Tricine-NaOH [pH 7.8], and 0.05% DM). Two green bands were obtained after centrifugation at 288,000 × *g* for 6 h in a swinging bucket rotor (Beckman, XPN-100, USA) at 4 °C, and the bands were collected with syringes and denatured at 60 °C for 10 min^36^.

### Measurements of ΔpH and Δψ

The two components of the *pmf* (ΔpH and Δψ) were measured via the ECS signal using a Dual-PAM-100 (Walz, Effeltrich, Germany) device according to the method described by Klughammer et al.^37^. The plants were fully dark adapted, after which they were subjected to 393 μmol m^−2^ s^−1^ of light, and the dark-interval relaxation kinetics were then obtained.

### Statistical analysis

Data gathered from at least three independently repeated experiments were analyzed via SPSS software by using Tukey’s test at the *P* < 0.05 level of significance.

## Supplementary information

The key cyclic electron flow protein PGR5 associates with cytochrome b6f and its function is partially influenced by LHCII state transition
