# Supplementary material for: The key cyclic electron flow protein PGR5 associates with cytochrome b6f, and its function is partially influenced by the LHCII state transition
Source: Hortic Res. 2021 Mar 4;8:55. doi: 10.1038/s41438-021-00460-y (PMC7933433; doi:10.1038/s41438-021-00460-y)
Supplement: Supplementary file 1 — The key cyclic electron flow protein PGR5 associates with cytochrome b6f and its function is partially influenced by LHCII state transition [file 41438_2021_460_MOESM1_ESM.pdf]

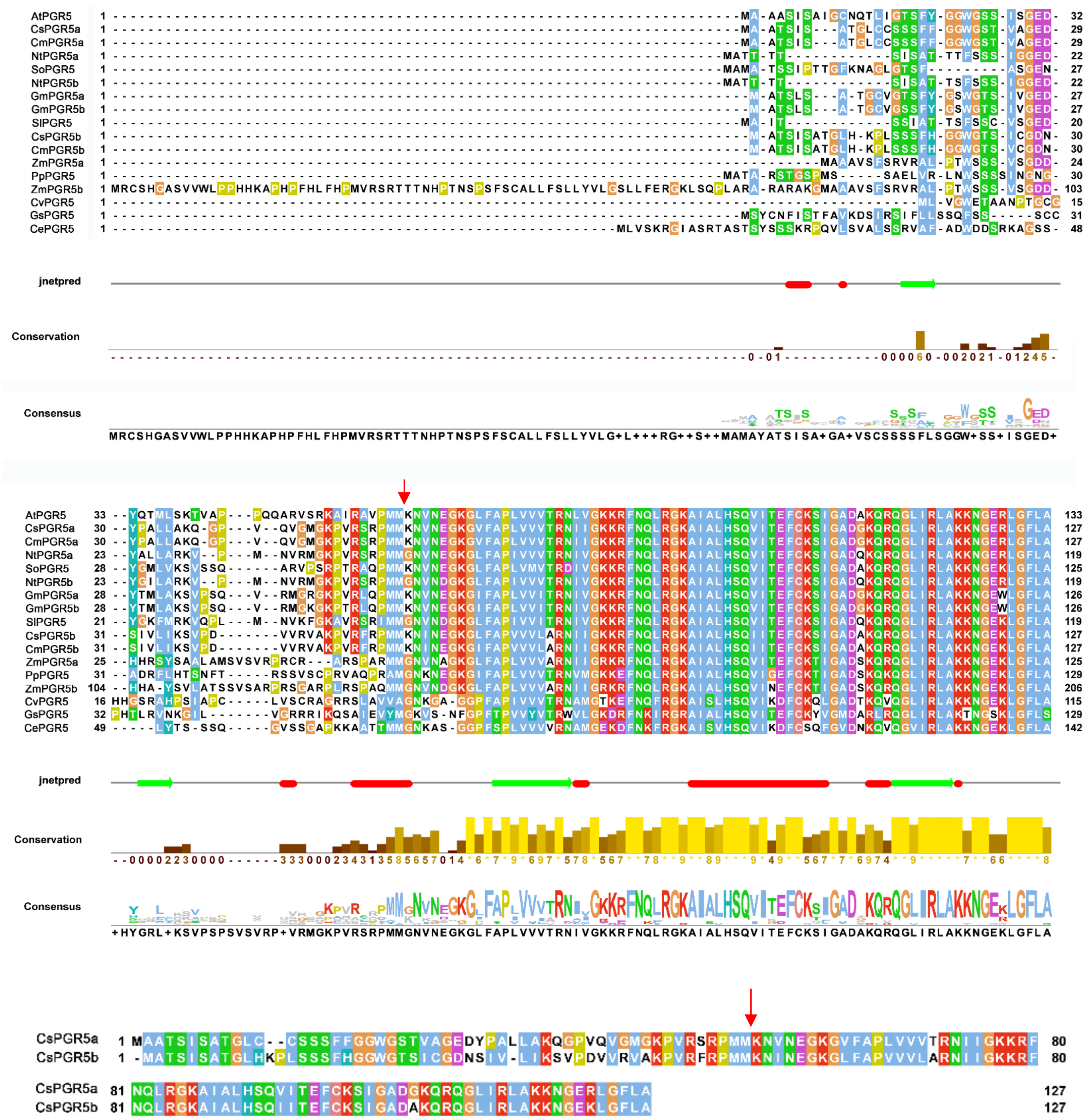

Fig. S1 Alignment of the amino acid sequences of PGR5 proteins among 11 species. *Arabidopsis thaliana* (At), *Cucumis sativus* (Cs), *Cucumis melo* (Cm), *Nicotiana tabacum* (Nt), *Glycine max* (Gm), *Solanum lycopersicum* (Sl), *Zea mays* (Zm), Physcomitrella patens subsp. Patens (Pp), *Chlorella variabilis* (Cv), *Galdieria sulphuraria* (Gs), *Chlamydomonas eustigma* (Ce). An arrow indicates the predicted cleavage site of the transit peptide.

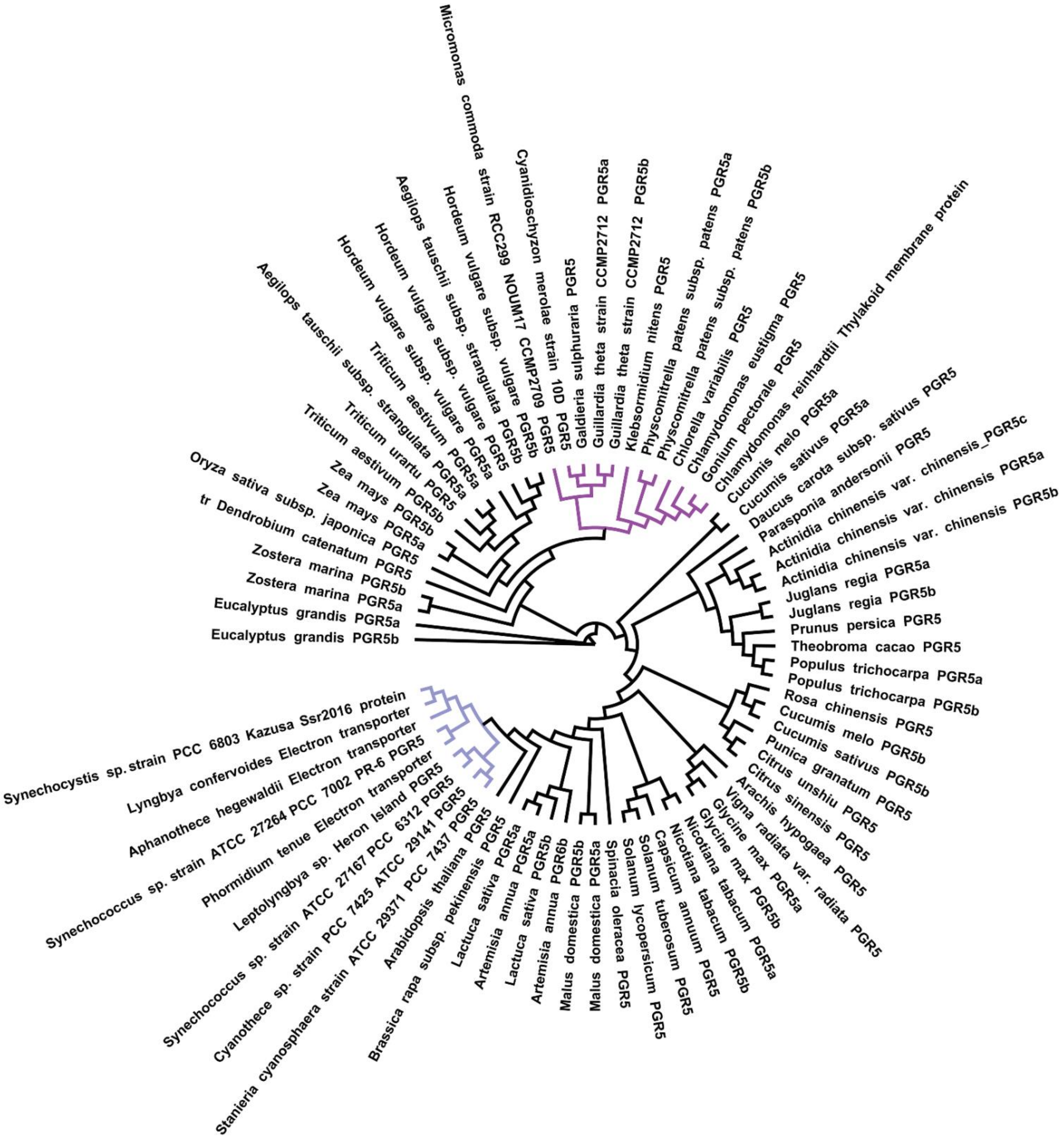

Fig. S2 Phylogenetic tree of PGR5 among 54 species. Amino acid sequences of CsPGR5a blast in NCBI were used to build the phylogenetic tree.

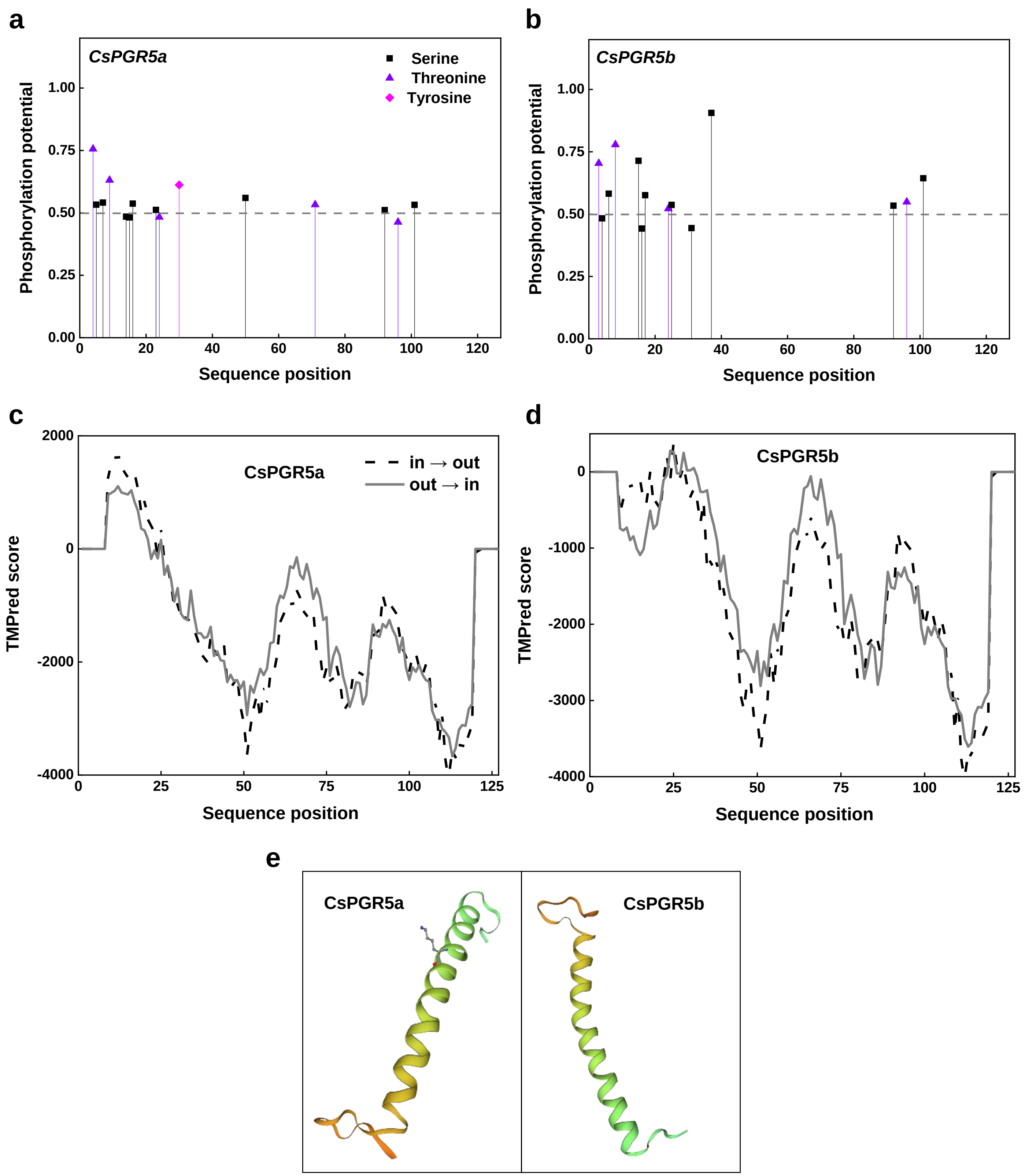

Fig. S3 The protein structure analysis of cucumber PGR5 proteins. **a, b** Phosphorylation sites analysis of CsPGR5a and CsPGR5b. **c, d** Prediction of the transmembrane domain of CsPGR5a and CsPGR5b. **e** Tertiary structure model on cucumber CsPGR5a and CsPGR5b proteins.

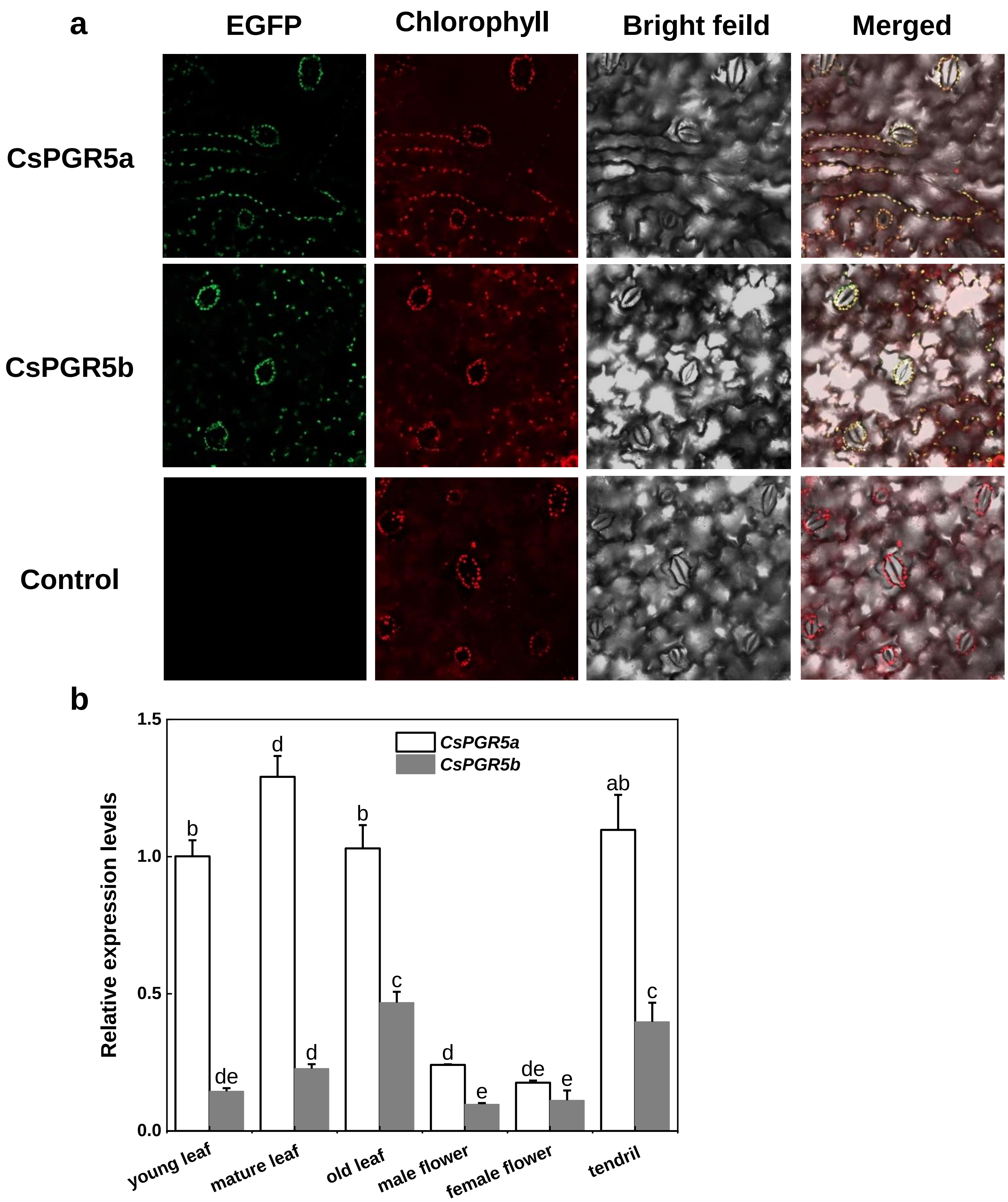

Fig. S4 Subcellular localization and expression patterns of cucumber PGR5 proteins. **a** The full-length sequence of *CsPGR5a* and *CsPGR5b* was fused with *GFP* and transiently expressed in tobacco leaves. **b** Gene expression patterns of *CsPGR5a* and *CsPGR5b* in various organs which were normalised to expression level of *CsPGR5a* in young leaf. Different letters indicate significant differences between treatments (P<0.05) according to Tukey's test.

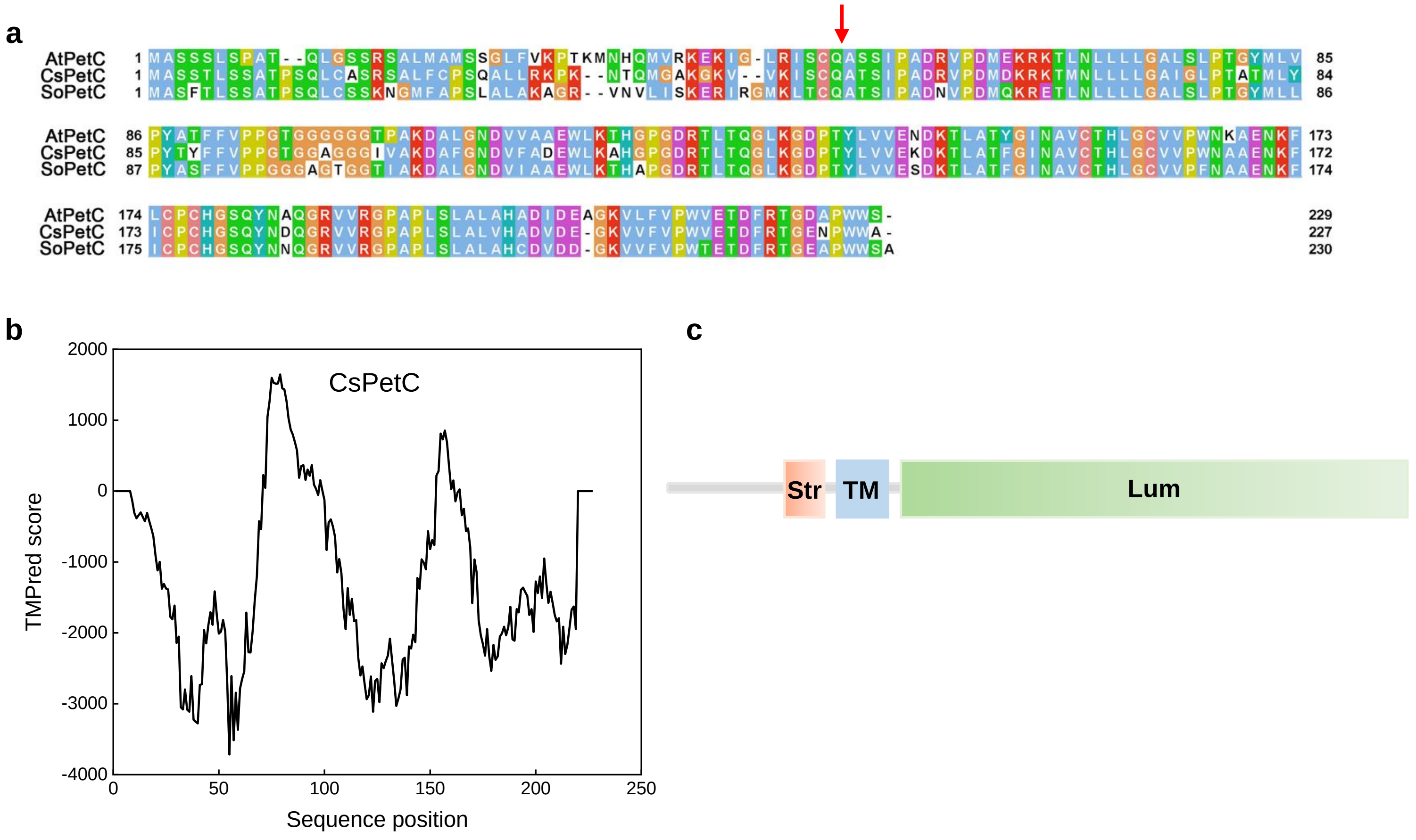

Fig. S5 Analysis of PetC protein. **a** Alignment of the amino acid sequences of PetC proteins among *Arabidopsis thaliana* (At), *Cucumis sativus* (Cs), *Spinacia oleracea* (So). An arrow indicates the predicted cleavage site of the transit peptide. **b** Prediction of the transmembrane domain of CsPetC. **c** The arrangement of the domains in CsPetC sequence, Str (stromal part); TM (transmembrane domain); and Lum (lumenal part).

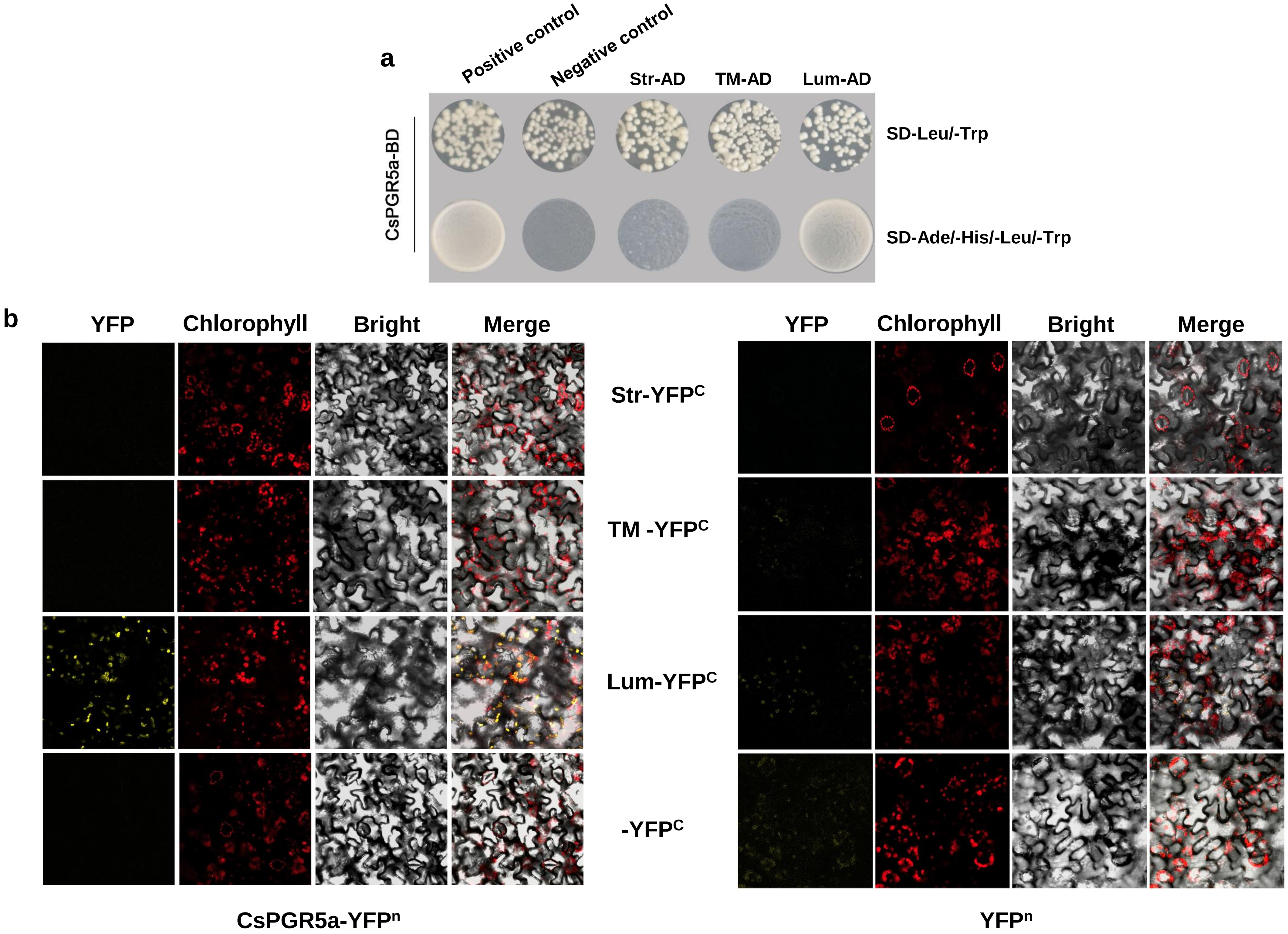

Fig S6 Analysis of the exact part of CsPetC interacted with CsPGR5a. The mature CsPetC was divided into three part, i.e. stromal part (Str), transmembrane (TM) domain, and lumenal part (Lum). **a** Yeast two-hybrid analysis. CsPGR5a was fused to the DNA-binding domain (PGR5a-BD), while the three parts of CsPetC were fused to the activation domain. pGBKT7-53 + pGADT7-T and pGBKT7-Lam + pGADT7-T were used as positive and negative controls, respectively. **b** BiFC assay. Plasmids encoding fusion constructs with the N- or C-terminal parts of YFP (PGR5a-YFP^{n}, Str-YFP^{c}, TM-YFP^{c}, and Lum-YFP^{c}) were separately transiently expressed in *N. benthamiana* leaves. Yellow signals indicate YFP fluorescence; magenta signals indicate chloroplast autofluorescence.

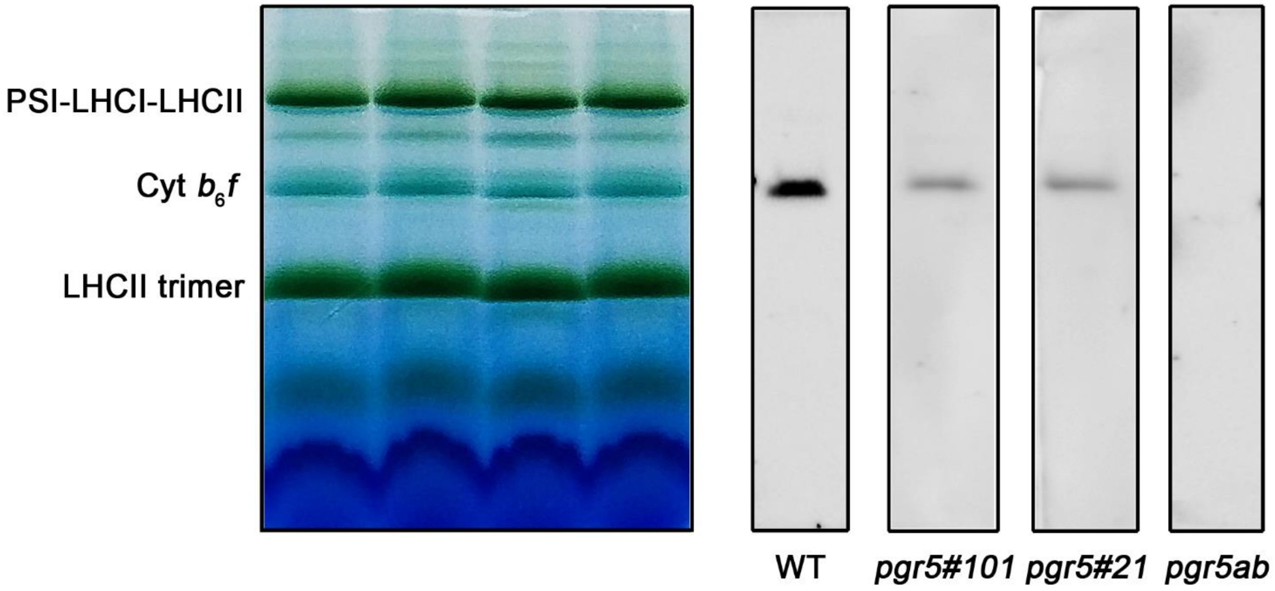

Fig. S7 BN-PAGE of tobacco's thylakoid membrane proteins in WT and *pgr5* mutants (*pgr5#101*, *pgr5#21*, and *pgr5ab*), anti-PGR5 antibody was used for immunoblotting.

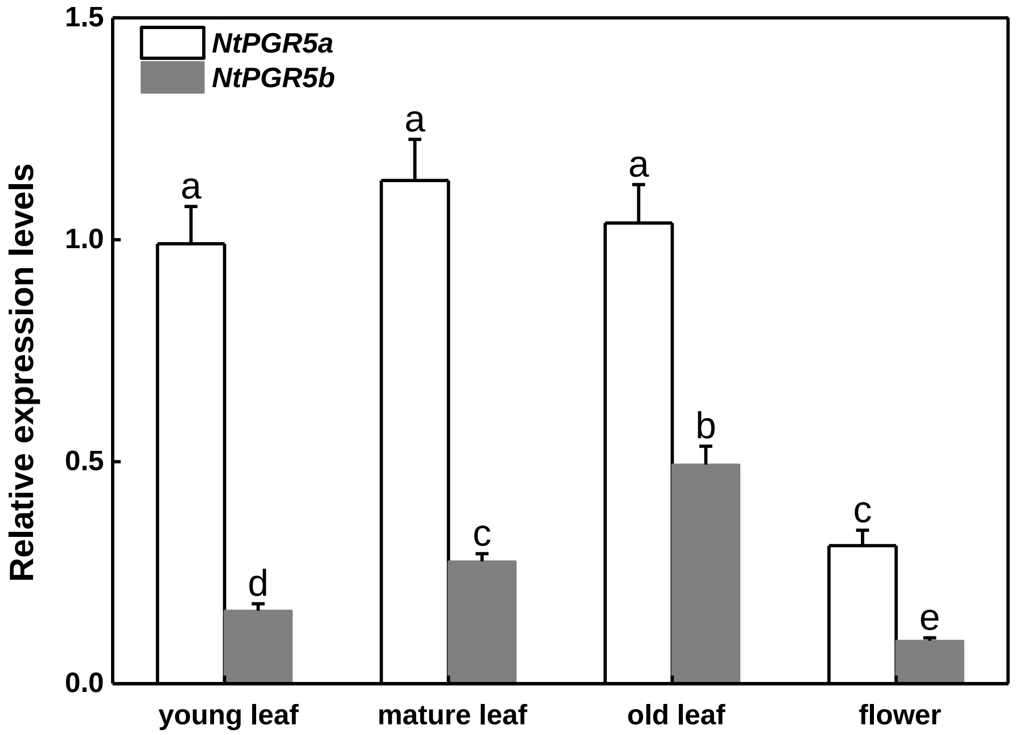

Fig. S8 Expression patterns of *NtPGR5a* and *NtPGR5b* in various organs. Different letters indicate significant differences between treatments ($P<0.05$) according to Tukey's test.

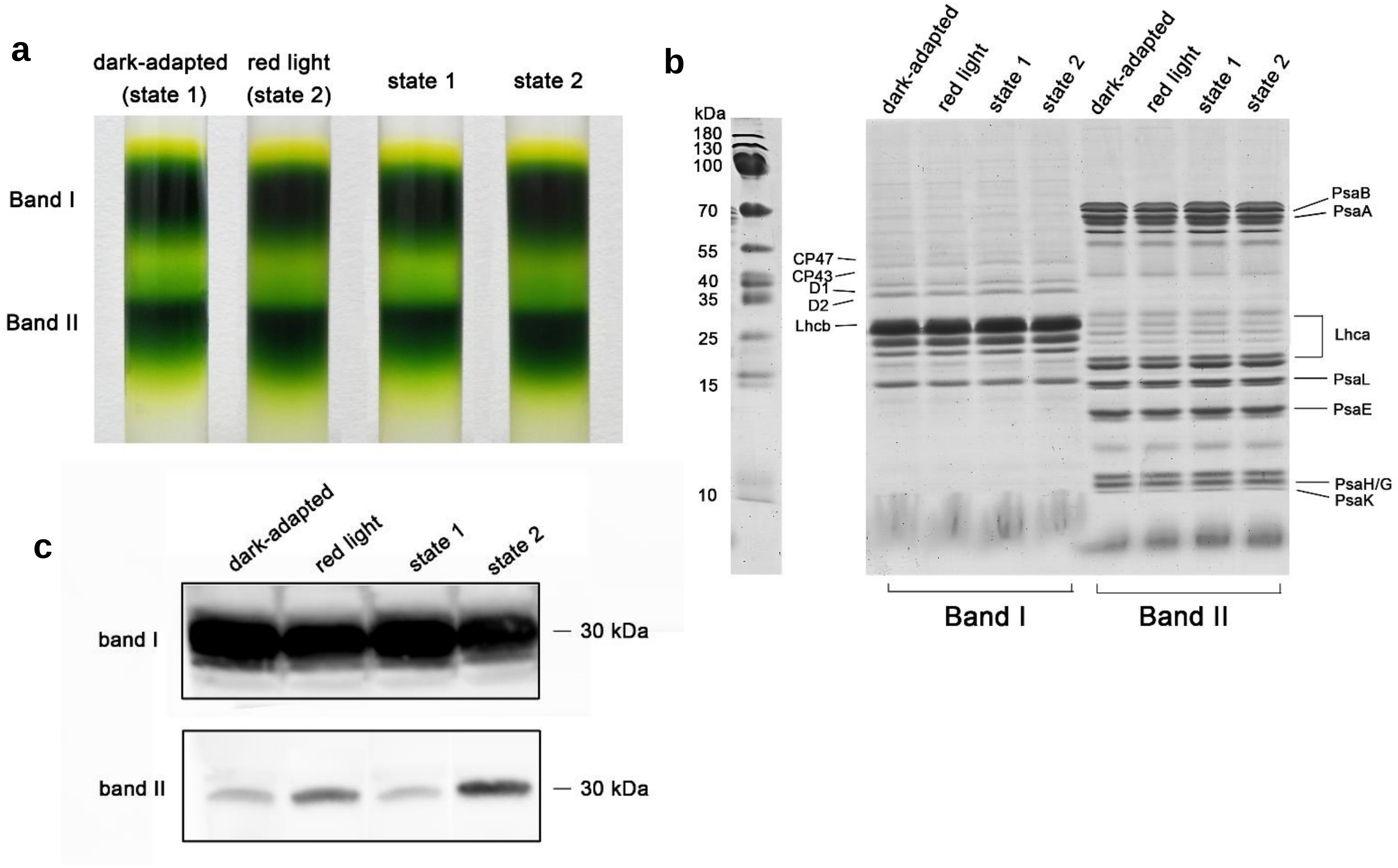

Fig. S9 Analysis of thylakoid membrane proteins under state transitions. **a** Separation of PSI- and PSII- supercomplexes by sucrose gradient ultracentrifugation in a swinging bucket rotor at 4 ℃. **b** SDS-PAGE analysis of the two bands separated from '**a**'. **c** Immunoblotting analysis by using anti-Lhcb1,2,3 antibody.

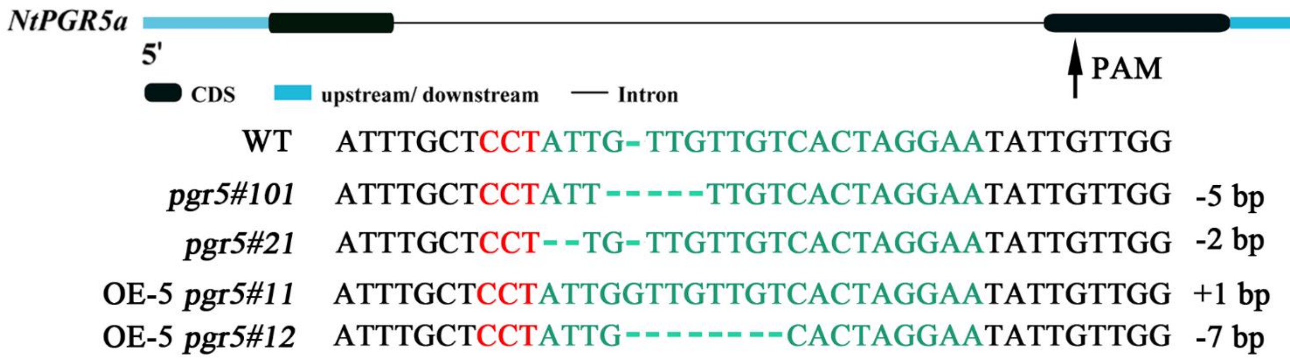

Fig. S10 Identification of *pgr5* mutants. DNA sequence comparison of WT, *pgr5#101*, *pgr5#21*, OE-5 *pgr5#11*, OE-5 *pgr5#12*. Sequencing analysis showed that *pgr5#101* mutant contained the 4 bp deletion in the exon, *pgr5#21* mutant contained the 2 bp deletion in the exon, OE-5 *pgr5#11* mutant contained the 1 bp insertion in the exon and OE-5 *pgr5#12* mutant contained a 7 bp deletion in the exon.

Table S1 Description of the cucumber *PGR5*

Gene name	Gene ID	Location in chromosome	CDS	aa	pI	MW
CsPGR5a	CsaV3_2G012500	2	384bp	127aa	10.45	13.48kDa
CsPGR5b	CsaV3_3G011780	3	384bp	127aa	10.87	13.72kDa

Table S2 The main *cis*-elements in the promoters of cucumber *PGR5* genes predicted PlantCARE

Gene	*Cis*-element name	motif sequence	Function	Number
CsPGR5a	ACE	GACACGTATG	light responsiveness	1
	CGTCA-motif	CGTCA	MeJA-responsiveness	2
	3-AF1 binding site	TAAGAGAGGAA	light responsive	1
	AE-box	AGAAACAA	light response	1
	I-box	cCATATCCAAT	light responsive	1
	Box 4	ATTAAT	light responsiveness	1
	TGACG-motif	TGACG	MeJA-responsiveness	2
	GTGGC-motif	GATTCTGTGGC	light responsive	1
	LAMP-element	CTTTATCA	light responsive	1
	CCAAT-box	CAACGG	MYBHv1 binding site	1
	L-box	ATCCCACCTAC	light responsive	1
	G-Box	CACGTT	light responsiveness	2
	G-Box	CACGTG	light responsiveness	2
	G-box	GCCACGTGGA	light responsiveness	1
	GT1-motif	GGTTAAT	light responsive	2
	GT1-motif	GGTTAA	light responsive	1
	ARE	AAACCA	anaerobic induction	3
	MBS	CAACTG	drought-inducibility	1
	TCA-element	CCATCTTTTT	salicylic acid responsiveness	1
	TCT-motif	TCTTAC	light responsive	1
	ABRE	ACGTG	abscisic acid responsiveness	3
	ABRE	CACGTG	abscisic acid responsiveness	1
CsPGR5b	GARE-motif	TCTGTTG	gibberellin-responsive element	1
	Box 4	ATTAAT	light responsiveness	2
	circadian	CAAAGATATC	circadian control	1
	GCN4_motif	TGAGTCA	endosperm expression	1
	GATA-motif	AAGGATAAGG	light responsive element	1
	O_2-site	GATGACATGG	zein metabolism regulation	1
	LAMP-element	CTTTATCA	light responsive	1
	ARE	AAACCA	anaerobic induction	5
	GC-motif	CCCCCG	anoxic specific inducibility	1
	TGA-element	AACGAC	auxin-responsive	1

Table S3 Candidate proteins interacting with CsPGR5a obtained from the yeast two-hybrid library

Number	Gene name	Gene ID	Protein name
1	PetC	LOC101221828	cytochrome b6-f complex iron-sulfur subunit, chloroplastic-like
2	LHCAP4	LOC101222701	chlorophyll a-b binding protein P4, chloroplastic
3	PsaH	LOC101223219	photosystem I reaction center subunit VI
4	PnsL5	LOC101218923	photosynthetic NDH subunit of lumenal location 5
5	atpB	LOC101205618	synthase subunit beta, mitochondrial-like
6	PP2C25	LOC101207841	probable protein phosphatase 2C 25
7	PsaK	LOC101209832	photosystem I reaction center subunit psaK, chloroplastic
8	Lhcb3	LOC101207278	chlorophyll a-b binding protein 13, chloroplastic CAB13
9	FDA	LOC101207278	chlorophyll a-b binding protein 13, chloroplastic CAB13
10	PsaG	LOC101218641	photosystem I reaction center subunit V, chloroplastic
11	CP12-2	LOC101220476	calvin cycle protein CP12-2, chloroplastic
12	PsbS	LOC101206477	photosystem II 22 kDa protein, chloroplastic
13	PGRL1B	LOC101207034	PGR5-like protein 1B, chloroplastic
14	PGRL1A	LOC101218313	PGR5-like protein 1A, chloroplastic
15	PGR5b	LOC101204671	protein PROTON GRADIENT REGULATION 5, chloroplastic

Table S4 Summary of LC-MS/MS analysis of the IP sample

Protein ID	Protein name	Gene name
B0F831	Proton gradient regulation 5 (PGR5a)	PGR5
A0A0A0L870	Uncharacterized protein (PGR5b)	Csa_3G134710
P32869	Photosystem I reaction center subunit II, chloroplastic	psaD
Q4VZN4	Photosystem I P700 chlorophyll a apoprotein A2	psaB
A0A343UM32	Photosystem I P700 chlorophyll a apoprotein A1	psaA
P42048	Photosystem I reaction center subunit III	PSAF
P42051	Photosystem I reaction center subunit psaK, chloroplastic	PSAK
A0A1X9Q1R5	Photosystem I iron-sulfur center	psaC
P42050	Photosystem I reaction center subunit VI, chloroplastic, PSI-H	PsaH
A0A218KG41	Cytochrome f	petA
A0A218KG87	Cytochrome b_6	petB
I7K435	Cytochrome b_6f complex iron-sulfur subunit	PetC
A0A0A0L3P3	Ferredoxin-NADP reductase, chloroplastic	Csa_3G002780
P08221	Chlorophyll a-b binding protein of LHCII type I, chloroplastic	Lhcb3
A0A0A0KXI3	Chlorophyll a-b binding protein, chloroplastic	Csa_4G308550
A0A0A0KXR5	Chlorophyll a-b binding protein, chloroplastic	Csa_5G646700
A0A0A0K565	Chlorophyll a-b binding protein, chloroplastic	Csa_7G033300
A0A0A0LCY3	Chlorophyll a-b binding protein, chloroplastic	Csa_3G664560
A0A0A0KEF5	Chlorophyll a-b binding protein, chloroplastic	Csa_6G057170
A0A0A0LXV0	Chlorophyll a-b binding protein, chloroplastic	Csa_1G630330
A0A0A0L6I8	Chlorophyll a-b binding protein, chloroplastic	Csa_3G099680
A0A0A0LSB4	Chlorophyll a-b binding protein, chloroplastic	Csa_1G009810
